# Properties and Therapeutic Potential of Transient Receptor Potential Channels with Putative Roles in Adversity: Focus on TRPC5, TRPM2 and TRPA1

**DOI:** 10.2174/138945011795378568

**Published:** 2011-05

**Authors:** L.H Jiang, N Gamper, D.J Beech

**Affiliations:** Institute of Membrane and Systems Biology, Faculty of Biological Sciences, University of Leeds, Leeds, LS2 9JT, UK

**Keywords:** Calcium-permeable channel, cationic channel, inflammation, pain, redox potential, remodeling, transient receptor potential.

## Abstract

Mammals contain 28 genes encoding Transient Receptor Potential (TRP) proteins. The proteins assemble into cationic channels, often with calcium permeability. Important roles in physiology and disease have emerged and so there is interest in whether the channels might be suitable therapeutic drug targets. Here we review selected members of three subfamilies of mammalian TRP channel (TRPC5, TRPM2 and TRPA1) that show relevance to sensing of adversity by cells and biological systems. Summarized are the cellular and tissue distributions, general properties, endogenous modulators, protein partners, cellular and tissue functions, therapeutic potential, and pharmacology. TRPC5 is stimulated by receptor agonists and other factors that include lipids and metal ions; it heteromultimerises with other TRPC proteins and is involved in cell movement and anxiety control. TRPM2 is activated by hydrogen peroxide; it is implicated in stress-related inflammatory, vascular and neurodegenerative conditions. TRPA1 is stimulated by a wide range of irritants including mustard oil and nicotine but also, controversially, noxious cold and mechanical pressure; it is implicated in pain and inflammatory responses, including in the airways. The channels have in common that they show polymodal stimulation, have activities that are enhanced by redox factors, are permeable to calcium, and are facilitated by elevations of intracellular calcium. Developing inhibitors of the channels could lead to new agents for a variety of conditions: for example, suppressing unwanted tissue remodeling, inflammation, pain and anxiety, and addressing problems relating to asthma and stroke.

## INTRODUCTION

Knowledge of the mammalian Transient Receptor Potential (TRP) channels arose from studies of *Drosophila melanogaster* TRP, which forms a non-selective cationic channel in photoreceptors and enables sustained responses to bright light. Searches of mammalian cDNA libraries and databases subsequently revealed an array of TRP-related proteins, now referred to as the mammalian TRP channels. There are known to be 28 *TRP*-related mammalian genes, most of which encode Ca^2+^-permeable non-selective cationic channels. Importance in human disease has been identified, for example through studies of polycystic kidney disease where TRP channel mutations are causative factors. TRP channels are thought to exist as homo- or hetero- tetramers, with the ion pore at the centre of the cluster. There appear to be six membrane-spanning segments per TRP protein and the N- and C-termini are intracellular. Based on amino acid sequence comparisons the channels are sub-divided into families that include, but are not limited to, the Canonical (C), Melastatin (M) and Ankyrin (A) types. In mammals there are 7 TRPC proteins, 8 TRPM proteins and 1 TRPA protein.

Much has been learned about mammalian TRP channels but the overall biological purpose of the channels remains a matter of debate. Unlike many other ion channels, TRP channels generally have weak voltage dependence and are not directly gated by major neurotransmitters, so they do not exist as primary determinants of electrical excitability or fast synaptic transmission. Instead, a hypothesis is that the TRP channels enable coupling of relatively slow chemical and physical events to cellular Ca^2+^-signalling systems, directly because of intrinsic Ca^2+^-permeability or indirectly through permeability to other ions, such as Na^+^. In several cases, events sensed by TRP channels are known. Some of the activating chemicals are dietary factors. Perhaps best known is capsaicin (of chilli pepper), which activates TRPV1 channels. There is also menthol activation of TRPM8 and carvacrol activation of TRPV3. All of these TRP channels are expressed in sensory neurones, supporting the persuasive hypothesis that TRP channels are players in the sensory systems of mammals. However, expression of the channels is not restricted to sensory neurones, in keeping with a general tendency for TRP channels to be expressed across many cell types of the body. Furthermore, TRP channels respond to a variety of other chemicals that are naturally present in the body and associated with physiology or pathology, including redox and lipid factors. TRP channels mostly do not show exclusive sensitivity to a single factor but rather sensitivity to activation by a range of chemicals, leading to a concept of TRP channels as polymodal chemical sensors, putatively serving as integrators of chemical signals throughout the body.

Here we review three mammalian TRP channels (TRPC5, TRPM2, TRPA1), which we have selected based on our research interests, new and intriguing knowledge of the channels, and an apparent common role of the channels in enabling cells and tissues to sense and respond to adversity. Aspects covered by the review include the properties and functional roles of the channels, potential of the channels as therapeutic drug targets, and current knowledge of the pharmacology. Other review articles cover the classification of TRP channels and details of TRP channels not addressed in this review, e.g. [1-4].

## 1 TRPC5

### 1.1 Tissue and Cellular Distribution

TRPC5 has been detected most readily in the brain, with broad but non-uniform expression across different brain regions [5-10]. It is also present in many other tissues of the body, but again it is not uniformly expressed. Non-neuronal examples of its expression include sperm head [11], vascular smooth muscle cells [12-14], endothelial cells [15-18], adrenal medulla [19], mammary glands [20], yolk sac [17], activated T cells [21], monocytes in hypertension [22], and cardiac ventricles in hypertension [23].

### 1.2 General Properties

TRPC5 is a functional plasma membrane ion channel [24]. Although it is active across the full range of physiological voltages, it is also voltage-dependent; voltage can be thought of as a modulator once the channel is activated by other mechanisms. Triple rectification may be evident as the voltage is changed from -100 to +100 mV but, most commonly, the signature current-voltage relationship (I-V) is dominated by double-rectification: outward and inward rectification coming together at an inflexion point near 0 mV. TRPC5 is largely devoid of a kinetic component when the voltage is changed rapidly, except for occasional decay when square hyperpolarizing steps are applied [25].

The unitary conductance of the TRPC5 channel is relatively large: 41 pS chord conductance was recorded at -60 mV [26]. There was a mean open time at -60 mV of 7.5 ms and frequency of opening of 6.2 Hz at 20-25 ºC [26]. The channel has similar permeability to Na^+^, Cs^+^ and K^+^ while lacking permeability to Cl^-^ [25,27-30]. Several divalent cations are permeant and Ca^2+^ permeability has been estimated to be at least twice that of Na^+^ [28,29]. Intracellular Mg^2+^ blocks TRPC5, reducing outward unitary current at +30 mV with an IC_50_ of ~0.5 mM [31]. Aspartic acid residue 633 is involved in the Mg^2+^ blockade. TRPC5 is inhibited by >100 µM extracellular gadolinium or lanthanum ion, whereas lower concentrations of lanthanides have striking stimulatory effects [26,28]. Effects of lanthanides may be stimulatory or inhibitory depending on the initial degree of TRPC5 activity prior to lanthanide exposure [32].

### 1.3 Endogenous Modulators

TRPC5 modulation is multi-factorial, complex and incompletely understood. The channel shows ‘versatility’ or ‘promiscuity’, with different stimulators able to converge on channel function. TRPC5, therefore, has capability to act as an integrative sensor that coordinates various signals at the level of Ca^2+^ entry. It may also show important constitutive activity.

A common stimulus for TRPC5 is an agonist at a G protein-coupled receptor. Many different receptors may be involved, including receptors for adenosine 5’-triphosphate, bradykinin, acetylcholine, histamine, IgM (B-cell receptor cross-linking), prostaglandin E2, thrombin, uridine 5’-triphosphate, sphingosine-1-phosphate, glutamate and cholecystokinin [8,10,13,28,33-36]. G protein activation is almost certainly a critical step down-stream of receptor-activation because agonist effects are partially mimicked by intra-cellular GTP-γ-S and inhibited by GDP-β-S [13,28,37]. G proteins of the G_q/11_ type have been implicated [37,38] but the requirement is not absolute because stimulation by sphingosine-1-phosphate is blocked by pertussis toxin, which shows a role for G_i/o _[13]. In human embryonic kidney cells, although acting via different G proteins, endogenous muscarinic receptors and sphingosine-1-phosphate receptors couple similarly to TRPC5 [13], suggesting either that G protein βγ is critical or that G_q/11_ α and G_i/o _a link via a com-mon pathway. The down-stream signal after the G protein is uncertain. An important role of phospholipase C (PLC) is implicated but the signaling is unlikely to be explained by a simple relationship between TRPC5 and PLC effects (changes in PIP_2_, IP_3_, Ca^2+^, diacylglycerol etc).

TRPC5 is also stimulated by activation of growth factor receptors, which do not signal through G_q/11_ or G_i/o_ [28,39]. In these cases, the channels seem not to be “activated” but trafficked as constitutively-active channels to the surface membrane. In human embryonic kidney cells, GFP-tagged TRPC5 was observed in punctate, non-endocytic, vesicles [28,39]. In response to epidermal growth factor (EGF) the punctae progressed from sub-plasma membrane to plasma membrane space over a period of 1-2 minutes [39]. EGF-evoked trafficking of TRPC5 was prevented by inhibitors of phosphatidylinositol (PI)-3-kinase or a dominant negative mutant of Rac1 [39]. The effector down-stream of Rac1 is suggested to be PIP-5-kinase because a dominant-negative mutant of this kinase was inhibitory. The proposed signaling cascade is EGF receptor, PI-3-kinase, Rac1 and PIP-5-kinase.

An unusual and striking feature of TRPC5 is stimulation by external lanthanides [25,26,28,36]. Acidic amino acid residues in the outer pore region (turret) are involved; particularly glutamic acid residue 543 at the top end of the fifth membrane-spanning segment [26]. The biological importance of the lanthanide effect is unknown. Humans contain lanthanides only at low concentrations and the ions are not known to have biological relevance. External Ca^2+^ mimics the lanthanide effect, but only at supra-physiological concentrations [26,27,36]. Recently it was reported that ionic lead (Pb^2+^) mimics the effect of lanthanides and that the effect depends on glutamic acid residue 543, leading to the hypothesis that TRPC5 may confer survival advantage by acting as a sensor of poisonous metal ions [40]. TRPC5 may also be potentiated by mild extracellular acidification [41]; relationship to glutamic acid residue 543 was also indicated, but TRPC5 channels carrying the double mutation E543Q/ E595Q unexpectedly retained acid sensitivity.

The TRPC5 turret is a target for thioredoxin, a protein that has mostly been studied in the intracellular context but which is also secreted. In its reduced form, extracellular thioredoxin stimulates TRPC5 channel activity by breaking a disulphide bridge in the turret near to E543 [32]. The data add to an emerging picture of the TRPC5 turret as a target for extracellular modulators and mechanism for coupling to the ion pore. The thioredoxin sensitivity of TRPC5 is part of a more complex system of redox modulation because there is stimulation of TRPC5-dependent Ca^2+^ entry by H_2_O_2 _[15]. Stimulation by nitric oxide has been suggested [15] but this result was not confirmed by a subsequent study [42].

Lysophosphatidylcholine has been identified as a stimulator of TRPC5, acting relatively directly [43]. Several other lysophospholipids are effective, including the important signaling lipid lysophosphatidic acid, but not lysophosphatidylethanolamine or phosphatidylcholine. Platelet-activating factor (PAF) and lyso-PAF are chemically similar to LPC and stimulate TRPC5. Sphingosine, sphingomyelin, ceramide and ceramide-1-phosphate (C1P) lack effect but sphingosylphosphorylcholine is a strong stimulator, and gangliosides and psychosine are modest stimulators. Cerebrosides, sulphatides, arachidonic acid and anandamide (an arachidonic acid metabolite) fail to stimulate. The data suggest a complex arrangement between TRPC5 activity and various lipid factors, supporting the hypothesis that a function of TRPC5 channels is to act as lipid signal transducers. The mechanisms of the effects vary from relatively direct effects in the case of lysophosphatidylcholine [43] to effects purely via G protein signalling in the case of sphingosine-1-phosphate [13] and the oxidized phospholipids POVPC and PGPC [44]. The ganglioside GM1 has been suggested to stimulate TRPC5 indirectly through α5β1 integrin [21,45].

Elevation of the intracellular Ca^2+^ concentration has a strong permissive role in TRPC5 function [28,29,36,46,47]. It has been suggested that intracellular Ca^2+^ may even be a direct agonist at TRPC5, making it a Ca^2+^-activated ion channel [48]. However, evidence for a direct effect is lacking because TRPC5 channels in inside-out patches have not been shown to be stimulated by Ca^2+^. Earlier studies showed that elevation of intracellular Ca^2+^ to 200 nM stimulated TRPC5 in the absence of an exogenous agonist [28,29,36] and TRPC5 expressed in *Xenopus laevis* oocytes was stimulated by ionomycin, an ionophore that evokes Ca^2+^-release [49]. However, these effects of Ca^2+^ were relatively small compared with those of other stimulators and may reflect facilitation of constitutive channel activity or channel activity evoked by endogenous agonists surrounding the cells or produced by the cells. High micromolar concentrations of Ca^2+^ are inhibitory and may contribute to desensitization [50,51].

TRPC5 has been observed to be stimulated by hypotonic extracellular solution (removal of mannitol) or physical pressure applied to the inside of the cell [52]. Stimulation has also been observed in response to store-depletion evoked by inhibition of the SERCA pump in intracellular Ca^2+^ stores [5,14,36,37,53]. The mechanisms of these effects are unclear and not all investigators have observed the effects (see [24]). It is conceivable that the effects arise indirectly, for example because G protein coupled receptors or lipid signaling pathways are stimulated.

### 1.4 Protein Partners

TRPC5 is one member of a family of seven mammalian TRPC channels. There is direct evidence that TRPC5 can assemble with its closest relative TRPC4, and TRPC1 [13,54]. There are indications that TRPC1 can associate with additional TRP channels but it is not clear if TRPC5 can also join these partnerships. Heteromultimeric TRPC5-TRPC1 channels have a different I-V from TRPC5 alone, with less inflexion and greater but not absolute linearity in the physiological range [29,32,39]. Unitary currents are almost ten times smaller compared with TRPC5 alone [29]. Similar regulation by receptor agonists, thioredoxin and lanthanides has been observed for TRPC5-TRPC1 compared with TRPC5 channels [13,29,32].

There is compelling evidence for association of TRPC5 with various Ca^2+^ sensing proteins, which include calmodulin, NCS-1, CaBP1, enkurin, and STIM1 [11,46,49,50,55, 56]. Calmodulin interaction occurs at the so-called CIRB (CaM-IP_3_ receptor binding) site [55]. Other protein partners of TRPC5 are Na^+^-H^+^ exchange regulatory factor [57], stathmins [58], the immunophilin FKBP52 [59], the dynamin superfamily member MxA [60], junctate [61], and the lipid binding protein SESTD1 [62].

### 1.5 Functions

Several studies support the conclusion that TRPC5 has a role in growth cone extension and axonal guidance, although there is divergence of conclusions on the polarity of the effect, which may be due to the stage in the process at which intervention occurred [9,45,46,58]. Similarly a positive role in vascular smooth muscle migration has been observed, whereas the effect on endothelial cell migration was inhibitory [13,16]. Podocyte migration was stimulated by TRPC5 (via Rac1) and inhibited by TRPC6 (via RhoA) [63]. It is clear, therefore, that TRPC5 activity influences cell movement or the movement of parts of cells. More detailed studies are needed to investigate the timings and spatial aspects of TRPC5’s roles in these processes and to elucidate down-stream pathways. Coupling to calmodulin kinases has been suggested [9]. The contribution to cell migration in the cardiovascular system may be important in cardiovascular remodeling and the metabolic syndrome [17,64].

Stimulation of TRPC5 heteromultimers was suggested to occur in response to cholecystokinin in neurons of the fear centre, the amygdala [8]. The suggestion was supported by studies of *Trpc5*-/- mice, which showed a lowered sense of innate fear [10] and raised the possibility that sensing of Pb^2+^ and H^+^ by TRPC5 enables the channel to have roles in stimulating awareness of toxic metal poisoning or suffocation. 

Other suggested roles of TRPC5 are in the regulation of matrix metalloprotease secretion from fibroblast-like synoviocytes in rheumatoid arthritis [32], degranulation of mast cells [65], neuron-protection against HIV-1 transactivator protein [66], and autoimmune suppression [21].

### 1.6 Therapeutic Potential

*Trpc5*-/- mice are approximately normal, which suggests that the *Trpc5* gene is not critical for life of a young laboratory mouse [10]. Putting aside the possibility of redundancy and functional compensation in heteromultimeric complexes, it may also be concluded that modulators of TRPC5 function would not be lethal, perhaps even in humans. Therefore, it is reasonable to consider whether exogenous modulators of TRPC5 might be useful in the treatment of disease. One possibility is that TRPC5 inhibitors could be useful as new anxiolytics, potentially complementing existing anxiolytics such as diazepam, or over-coming problems associated with current agents. Another possibility is that TRPC5 inhibitors might be useful to suppress unwanted cardiovascular remodeling, including within the context of hypertension and the metabolic syndrome. Effects on neuronal growth cones and axonal guidance are possible reasons for caution, particularly in developmental contexts. There might also be unwanted effects on the immune system. The net balance of effects would almost certainly depend on the specific heteromultimeric arrangements of TRPC5 in different contexts and on the channel’s activity relative to other mechanisms in different conditions.

### 1.7 Pharmacology

Externally-acting anti-TRPC5 blocking antibody has been developed by two independent groups [18,32,53]. Although no specific or potent exogenous chemical inhibitors of TRPC5 are known, various chemicals have effects on TRPC5 function. In many of these cases it is not clear if the agent acts directly at TRPC5. TRPC5 has been reported to be inhibited by 25 µM SKF-96365 [27], 0.1-10 µM 3,5-bis(tri-fluoromethyl)pyrazole derivative BTP-2 [67], 100 µM flufenamic acid [38], the calmodulin inhibitors 100 µM W-13 or chlorpromazine [68], 100 µM W-7 or 5 µM calmida-zolium [69], 0.3 µM Pyr2 [70], 20 µM 2-aminoethoxy-diphenyl borate [25], and the myosin light chain kinase inhibitors 3 µM ML-7 or ML-9 [68,69]. We have not confirmed that BTP-2 inhibits TRPC5, instead we find slight stimulation (M Clynes and DJ Beech, unpublished data). Stimulation of TRPC5 by 50 µM genistein or diadzein was recently reported [18]. TRPC5 has been found to be resistant to 10 µM U73343, 30 µM dihydrosphingosine, 10 µM staurosporine, 1 µM bisindolylmaleimide I, 10 µM genistein, 10 µM wortmannin (but see [68]), 1mM sodium orthovanadate, 300 µM indomethacin or 50µM RHC-80267 [28], and 1 µM nifedipine, 10 µM methoxyverapamil, 25 µM berberine [25] or 100 µM MRS-1845 [25]. 

## 2 TRPM2

### 2.1 Tissue and Cellular Distribution

TRPM2 exhibits widespread tissue and cellular distribution with mRNA being abundant in the brain and detectable in immune and many other tissues and cell types [71-75]. In addition, TRPM2 channel activities have been documented in a diversity of cells, including neurons [76-80], microglia [75,81], pancreatic β-cells [82, 83], endothelial cells [84], and immune and other blood cells [72,85-94]. Many studies have focused on TRPM2 as a plasma membrane channel, but there is a report suggesting that it also exists as a lysosomal channel in pancreatic β-cells [82].

### 2.2 General Properties

TRPM2 channels are assembled as homomers directed by a C-terminal coiled-coil domain [95]. A special molecular feature of TRPM2 is that the distal C-terminal tail shows strong homology to the NUDT9 proteins, exhibiting adenosine 5’-diphosphoribose (ADPR) pyrophosphatase activity. Although the role of the enzymatic activity is unclear, the domain provides the site for ADPR binding and thereby confers activation of the TRPM2 channels by ADPR [72]. The TRPM2 channels are permeable to all physiological cations including Ca^2+^ and activation leads to increases in intracellular Ca^2+^ concentration ([Ca^2+^]_i_) and/or membrane depolarization [72,85,96]. The current-voltage (I-V) relationship exhibits straight linearity. The single channel conductance is typically 50-80 pS [72,78,85,88,97,98]. In addition to the full-length (TRPM2-L), several splicing variant isoforms are identified [74,99] including TRPM2-S, which contains the N-terminus and the first two transmembrane segments. This truncated isoform does not form functional channels, but imposes dominant negative inhibition of the TRPM2-L [100].

### 2.3 Endogenous Modulators

In addition to ADPR, nicotinamide adenine dinucleotide (NAD) and its metabolites including 2’-O-acetyl-ADPR, cyclic ADPR (cADPR) and nicotinic acid-adenine dinucleotide phosphate (NAADP) activate the TRPM2 channels. The EC_50_ for ADPR effects at TRPM2 is 10-90 µM. 2’-O-acetyl-ADPR, a metabolite of the SIR2 protein deacetylases, shows similar effectiveness [101]. The EC_50_ values for NAD, NAADP and cADPR are 1-1.8 mM, 0.73 mM and 0.7 mM, respectively, which are higher than the physiological concentrations [73,85,86,102,103]. However, these activators show remarkable synergy with ADPR. AMP, an ADPR metabolite, has no agonist activity but inhibits the channel activation by ADPR with an IC_50_ of 70 µM [86,102].

The TRPM2 channels can also be activated by oxidative stress stress such as H_2_O_2_ [73,99,100]. The activation by H_2_O_2_ is slow, typically taking several minutes. The underlying molecular mechanism is still under investigation. Although there is evidence supporting direct gating [99], more recent studies suggest an indirect activation mechanism through activating poly(ADPR)polymerase (PARP)/ poly(ADPR) glycohydrolase (PARG) or NAD glycohydrolase (NADase) pathways to produces ADPR [84,91,104-106]. This latter mechanism is thought to also mediate activation of the TRPM2 channels by tumour necrosis factor- α (TNF-α) [73,90,107] and amyloid β-peptide [77]. Thus, expression of TRPM2 channels confers on cells an ability to sense and respond to changes in cellular redox status.

Ca^2+^ is essential for full activation of the TRPM2 channels; intracellular Ca^2+^ potently facilitates the channel activation by ADPR, or even directly activates the channels [108-110]. TRPM2 channel activity is enhanced by warm temperature of >40°C [82]. In contrast, extracellular or intra-cellular acidification inhibits the TRPM2 channel [94,98]. 

### 2.4 Protein Partners

Like many other ion channels, TRPM2 channels are functionally regulated by interacting proteins; calmodulin and protein tyrosine phosphatase PTPL1 have been so far identified. The calmodulin-TRPM2 interaction is Ca^2+^-dependent and enables intracellular Ca^2+^ to gate the TRPM2 channels [109-111]. PTPL1 is a protein tyrosine phosphatase; over-expression of PTPL1 reduces, whereas suppression of the PTPL1 expression increases, the phosphorylation level of the TRPM2 protein by unidentified tyrosine protein kinases and the TRPM2 channel-mediated responses to H_2_O_2_ and TNF-α [90]. 

### 2.5 Functions

Functional roles of TRPM2 have started to emerge, including effects on insulin release, cytokine production, endothelial permeability, and apoptotic/necrotic cell death. Togashi *et al.* showed that pancreatic β-cells respond to warm temperature with increased [Ca^2+^]_i_ and insulin release through activating the TRPM2 channels [81]. TRPM2 channels are also involved in insulin release stimulated by high levels of glucose via a K_ATP_ channel-independent mechanism [82]. 

Several cellular functions of the TRPM2 channels relate to oxidative stress (i.e. generation of H_2_O_2_). For instance, a recent study provided compelling evidence for a key role of TRPM2 in H_2_O_2_-induced increases in [Ca^2+^]_i_ that are essential in signaling cascades responsible for production of the chemokines CXCL8/CXCL2 in monocytes [92]. A separate study showed that H_2_O_2_ as well as ADPR induces Ca^2+^ influx in human pulmonary artery endothelial cells. H_2_O_2_ reduces trans-monolayer endothelial electrical resistance in a concentration-dependent manner. Such H_2_O_2_-evoked effects were enhanced by over-expressing TRPM2-L, or attenuated by over-expressing TRPM2-S to inhibit the endogenous TRPM2 channel function, or using PARP inhibitors to block ADPR formation [84]. These results suggest an important role for the TRPM2 channels in mediating H_2_O_2_-induced impairment of the endothelial barrier functions. The most widespread functional role of the TRPM2 channels is, perhaps, to mediate oxidative stress-induced cell death. This has been consistently demonstrated in cells expressing the recombinant and endogenous TRPM2 channels, including neurons, monocytes, lymphocytes, insulin-secreting cells and cardiomyocytes [73,76,77,87,90,100,101,106,107,111-113]. TRPM2 channels also mediate cell death induced by TNF-α, Aβ_42_, concanavalin A and puromycin (a pleiotropic cell stress agent) [73,77,87,90,101,107]. The importance of the TRPM2 channels in cell death induced by H_2_O_2_ and other cytolytic stimuli is supported by the observations that the cell death is attenuated by reducing the expression and function of endogenous TRPM2 channels or preventing ADPR formation [73,77,87,90,100,104,107].

Lipopolysaccharide (LPS) and TNF-α stimulate the generation of reactive oxygen species (ROS) in immune cells. A more recent study has shown that LPS and TNF-α significantly up-regulate TRPM2 expression and enhance ADPR-induced currents in human primary monocytes, resulting in elevated basal [Ca^2+^]_i_ and production of IL-6, IL-8, IL-10 and TNF-α. These effects are reduced when TRPM2 expression is suppressed by RNA interference [93]. A separate study has implicated a role for TRPM2 in prostate cancer cell proliferation [114].

### 2.6 Therapeutic Potential

The suggested roles of TRPM2 in insulin secretion from pancreatic β-cells [82] and chemokine production in monocytes [92] imply that alteration in expression and function of TRPM2 may increase susceptibility to diabetes and inflammatory disease. Indeed, *Trpm2* gene ablation reduces chemokine expression, neutrophil infiltration, and ulceration in a colitis animal model [92]. The finding that the TRPM2 channels mediate oxidative stress-induced endothelial hyperpermeability recognises TRPM2 channels as an important factor in vascular barrier dysfunction of cardiovascular disease [115]. 

There is also accumulating evidence to support a role for the TRPM2 channels in the pathogenesis of neurodegenerative disorders, which share common features including prominent disruption in Ca^2+^ homeostasis triggered by oxidative stress. Loss of neuronal cells due to activation of the TRPM2 channels by oxidative stress, TNF-α and Aβ_42_ strongly suggests a role of the TRPM2 channels in the patho-physiology of Alzheimer’s [77,78,116]. Altered TRPM2 channel expression and/or function are also reported under diseased conditions such as stroke, Western Pacific amyotrophic lateral sclerosis (WP-ALS) and parkinsonism-dementia (PD). For example, TRPM2 expression is up-regulated in microglia that parallels with microglial activation in a stroke animal model, consistent with the idea that the TRPM2 channels in microglia are involved in the CNS responses to oxidative stress and brain damage due to ischemic injury [77]. However, intriguingly, a recent study has identified a mutation (P1018L) in WP-ALS and PD patients, which is located in the pore loop of the TRPM2 channel and introduces fast channel inactivation [117]. 

### 2.7 Pharmacology

The pharmacology of the TRPM2 channels is currently limited. Nonetheless, several compounds have been identified that inhibit the TRPM2 channels, including 8-Br-cADPR, flufenamic acid (FFA), imidazole anti-fungal agents (clotrimazole and econazole), N-(p-amylcinnamoyl) anthranilic acid (ACA), and 2-aminoethoxydiphenyl borate (2-APB). One study found no effect of 2-APB on TRPM2, possibly because of a pore-dilation effect in the channels [25]. 8-Br-cADPR strongly suppresses the channel activation by cADPR, NAD, NAADP and H_2_O_2_, but intriguingly shows significant synergy with ADPR [85,99]. FFA, a non-steroidal anti-inflammatory metabolite, completely inhibits the TRPM2 channels in a concentration-independent (50-1000 µM) and largely irreversible manner [118]. Clotrimazole and econazole in the range examined of 3-30 μM display virtually the same actions as FFA [119]. In contrast, ACA and 2-APB display a reversible and concentration-dependent inhibition with IC_50_ of 1.7 µM and 1.2 µM, respectively [120,121]. It should be emphasized that all these inhibitors cause well-documented inhibition at a wide spectrum of ion channels, receptors and enzymes [118,120,121]. Therefore, potent and specific TRPM2 channel inhibitors are still required for elucidation of the functional roles of the TRPM2 channels as well as for the purposes of therapeutic exploitation.

## 3 TRPA1

### 3.1 Tissue and Cellular Distribution

TRPA1 mRNA was reported in different mammalian tissues, including brain [122,123], intestine and pancreas [123]. However, functional activity of TRPA1 channels is most consistently characterized in sensory neurons and other cells with sensory functions. TRPA1 is present in subsets of peripheral sensory neurons of dorsal root (DRG), trigeminal (TG) and nodose ganglia [124-126]; these TRPA1-positive sensory neurons belongs to C-fibres and usually also express nociceptor markers TRPV1, calcitonin gene related peptide (CGRP), substance P but not IB-4 [124,125,127, 128]. TRPA1 is expressed in vestibular and auditory sensory epithelia such as mechanosensory hair cells of the inner ear in mammals [124,129] or of lateral line in fish [130] where it is suggested to participate in mechanotransduction. High level of TRPA1 expression is also reported in skin keratinocytes [128,131] and there is expression in the enterochromaffin cells of the gastrointestinal tract [132]; these cells stores serotonin (5-hydroxytryptamine; 5-HT) and are believed to be able to respond to the chemical composition of the gut lumen by 5-HT release and thus regulate gastrointestinal contractions. Functional TRPA1 has been reported in synoviocytes of joints [133] and endothelial cells [134].

### 3.2 General Properties

TRPA1 channels are permeable to mono- and divalent cations and have a single channel conductance in the range of 100 pS (reviewed in [122]). At room temperature the monovalent cation permeability sequence for TRPA1 was estimated to be Rb^+^ ≥ K^+^ > Cs^+^ > Na^+^ > Li^+^ [135]. Permeability of TRPA1 to Ca^2+^ is higher than to Na^+^ with *P*_Ca_/*P*_Na_ close to 6 under basal conditions (no agonist stimulation); the fractional Ca^2+^ current in the presence of physiological concentrations of ions was estimated to be in the range of 17 % [135]. Based on the permeability to cations of different size, the pore diameter of TRPA1 under basal conditions was estimated in the range of 11Å. An interesting feature of several thermo-TRP channels is the phenomenon of pore dilation: upon agonist stimulation (sometimes a prolonged stimulation is required) the pore of these channels increases its size becoming permeable to large organic molecules such as NMDG, spermine, Yo-Pro, gentamycin etc. (reviewed in [136]). TRPA1 also undergoes pore dilation upon stimulation with mustard oil [135,137]. It was estimated that in the dilated state the TRPA1 pore diameter increases by approximately 3Å [135].

### 3.3 Endogenous Modulators

TRPA1 is a polymodal channel that can be stimulated by distinct mechanisms: *i)* covalent modification of cysteine and lysine residues within the N-terminus of the channel [138,139]; *ii)* non-covalent lock-and-key interaction with ligands (e.g. icilin, d-9-tetrahydrocannabinol, nicotine) [138-141]; *iii)* elevated intracellular Ca^2+^ and other intermediates of G protein coupled receptor (GPCR) cascades [142]; *iv)* cooling below 15^o^C (the matter is under debate; for recent reviews see [143,144]; *v)* depolarisation [145]. 

Probably the most important mechanism of TRPA1 activation, which also underlies its most substantiated physiological function (i.e. sensing irritants) is activation through covalent modifications of cysteine and perhaps also lysine residues in the N terminus of the channel. Although activity of many ion channels can be modified by cysteine modifications, TRPA1 is striking in the range of compounds and types of cysteine modifications it is responsive to. The compounds include plant-derived pungent and irritant chemicals such as allyl isothiocyanate (mustard oil, MO), thio-sulfinate (onion), α,β-unsaturated aldehydes (cinnamon) [138], air pollutants, cigarette smoke components (alorecin, nicotine), tear gas components (chlorobenzylidene malononitrile), formaldehyde, reactive oxygen species (ROS), chlorine and many others [138,139,146,147]. A general property of these compounds is that they contain highly reactive electrophilic carbon atoms which can react with cysteines forming reversible covalent modifications or adducts [138,139,148]. TRPA1 can also be irreversibly modified by classical cysteine-modifying reagents such as N-metyl maleimide (NMM) and (2-aminoethyl)methanethiosulphonate (MTSEA) [138,139]. In addition, direct activation of TRPA1 by ROS (which also commonly promote disulfide bonds between cysteines or formation of reversible cysteine modification to cysteine sulfenic acid [149] has also been reported [150]. Two groups found five cysteines (all in the channel N-terminus) to be responsible for TRPA1 activation by irritants but only one of them (C622 in mouse or C619 in human TRPA1) was identified by both groups [138,139]. Covalent modification of lysine 708 (in human TRA1) by isothiocyanates was also reported to contribute to activation of TRPA1 [138] (although in another study lysine-modifying agents did not activate TRPA1 [139]). Structural background of the TRPA1 gating by covalent modification within the N-terminus remains to be elucidated although a recent study indicated that the TRPA1 pore region is important for the coupling between covalent modifications of N-terminal cysteines and channel gating [151]. Recent study has demons-trated that the irritant-sensing function of TRPA1 is evolutionarily conserved and is already evident in insects [152]. 

TRPA1 is directly activated by intracellular Ca^2+^ and it was suggested that Ca^2+^ can directly bind to the N-terminal EF-hand domain of TRPA1 [142,153]. Ca^2+^ sensitivity of TRPA1 was recently suggested to underlie activation of TRPA1 by cold. Thus, it was suggested that, in HEK293 cells, cold-induced Ca^2+^ influx through unidentified channels (but not cooling itself) activates TRPA1 which otherwise has no intrinsic cold sensitivity [142]. This issue is still actively debated (see below). Activation of TRPA1 by Ca^2+^ may also underlie suggested activation and/or sensitization of TRPA1 by GPCRs coupled to PLC signalling, such as bradykinin receptors B_2_ and protease activated receptors PAR-2 [146,154,155]. Several other intermediates of the PLC signalling cascade such as PIP_2_ [155], diacyl glycerol and arachidonic acid [154] have been implicated in the action of PLC-coupled receptors and inflammatory mediators but this, again, is a controversial area and while sensitization (i.e. increased sensitivity to agonists) of TRPA1 by PLC-coupled receptors is reproducible in many studies, the acute activation of TRPA1 by these receptors is not; thus, in TRPA1 knock-out mice bradykinin induced similar acute excitation of C-fibres as in wild-type animals [156]. In addition, Liu *et al*. found little evidence for activation of any ruthenium red-sensitive TRP channels by bradykinin in cultured DRG neurons [157]. Ca^2+^ was also implicated in TRPA1 desensitisation [124,158], which further complicates the relationship between GPCR signaling and TRPA1 activity.

Despite its original identification as ion channel activated by noxious (<15^o^C) cold [126,154], the cold-sensitivity of TRPA1 is still debated. Two groups were unable to record activation of heterologously expressed TRPA1 by cold [124,159], another group suggested that cold sensitivity of the channel is due to its Ca^2+^ sensitivity [142]. In addition, poor correlation between cold- and MO-sensitivity of cultured nociceptive neurons and inconclusiveness of behavioural experiments on *Trpa1*^-/-^ mice ([160] *vs*. [146] *vs*. [145]) further contributed to the controversy. A recent study presented new evidence for cold sensitivity of TRPA1 [145]: the authors demonstrated that heterologously expressed TRPA1 channels can be activated by cold temperature in Ca^2+^-free solutions; additional behavioural tests on *Trpa1*^-/-^ mice (cold plate and tail flick) highlighted reduced sensitivity of transgenic animals to very cold temperatures (0 and -10 ^o^C). The authors argued that cold-induced Ca^2+^ transients in TRPA1-positive neurons are slow (~100 s to full effect in Ca^2+^ imaging paradigm) and rather small, which may be why these effects were overlooked in other studies. It remains unclear whether the slow kinetics of TRPA1 activation by cooling is compatible with relatively fast (~5s) onset of nocifensive behaviour in mice responding to cold [145].

As other thermo-TRP channels, TRPA1 is weakly voltage-sensitive with a calculated gating charge (*z*) of 0.375 [145]; for comparison, gating charge in a Shaker voltage-gated K^+^ channel is more than 12 [161]. The structural background for voltage-dependence of TRPA1 is unclear. In contrast to the voltage sensor domain of Kv channels, which has been localised to transmembrane segment S4 and is characterized by an array of positive charges, S4 of TRPA1 does not have any positive charges [136]. There are currently two main hypotheses describing the gating of thermo-TRP channels by voltage and temperature. One hypothesis is that the voltage-dependence is a fundamental principle of the channel gating and other stimuli, such that temperature and agonists affect channel gating by shifting the voltage dependence [145,162]. Another hypothesis involves an allosteric model where it is assumed that the temperature and voltage sensors are independent structures coupled to channel gating [136,163]. 

As for many other ion channels, there are reports that TRPA1 interacts with and can be regulated by the plasma membrane phospholipid PIP_2_, although the nature of this interaction is controversial since some groups report that PIP_2_ directly inhibits or desensitizes TRPA1 [155,164] while others suggest that PIP_2_ is required for TRPA1 activity and thus activates the channel [158,165].

### 3.4 Protein Partners

There is evidence that in sensory neurons and expression systems TRPA1 can interact (at least functionally) with TRPV1. The conclusions are based on data suggesting: i) that cannabinoid-induced dephosphorylation of TRPV1 requires functional TRPA1 [166]; ii) a phenomenon of cross-desensitization of TRPV1 and TRPA1 responses [167,168]; iii) co-expression of TRPA1 and TRPV1 in sensory neurons and expression systems results in whole-cell and single-channel currents with properties that cannot be adequately described by independent co-expression of TRPA1 and TRPV1 [169,170]; iv) TRPV1 and TRPA1 can be co-immunoprecipitated from neurons and expression system [170]. It is, however, still unclear if TRPV1 and TRPA1 can form *bona-fide* heteromultimers.

TRPA1 possesses an extended and highly-structured N-terminus harbouring large numbers of ankyrin repeats, which suggest a rich background for interactions with other molecules; surprisingly, the information on such interactions is largely missing.

### 3.5 Functions

TRPA1 is a non-selective cation channel which upon activation conducts depolarising currents and may, therefore, trigger action potentials (APs). Since TRPA1 is expressed in populations of sensory neurons and other cell types with sensory functions, it qualifies for a role of bodily sensor for any stimulus capable of providing enough *in vivo* TRPA1 activation to trigger AP firing.

As discussed, the role of TRPA1 as a noxious cold sensor is still debated. Likewise no consensus is reached about the role of TRPA1 in mechano-transduction: despite high TRPA1 expression in the mechano-sensitive hair cells, mechano-sensitivity of these cells is normal in TRPA1 knock-out mice [144,146] and zebrafish [130] (a recent report however provided evidence that mechano-sensitivity of colonic afferents is deficient in *Trpa1* knock-outs [156]). Most researchers are in agreement however that one of the major functions of TRPA1 is sensitivity to environmental irritants. In accord with this function, TRPA1 is expressed in sensory neurons innervating skin, airways and gastrointestinal tract and thus has a broad interface for interaction with different airborne compounds and components of the food.

The presence of functional TRPA1 in nociceptive neurons of the dorsal root and trigeminal ganglia suggests a role for these channels in pain because APs generated by these, normally silent, neurons is a first step in nociceptive transduction that can lead to the sensation of pain. Accordingly, a recent study has identified a gain-of-function mutation (N855S) within S4 of TRPA1 as a cause of a rare human condition - heritable episodic pain syndrome [171]; this disorder is characterised by episodes of debilitating pain triggered by fatigue, fasting, and cold. In addition, inflammation and tissue injury often trigger local production of electrophilic compounds such as ROS, which can directly activate TRPA1 and produce inflammatory pain. Recently a pro-inflammatory electrophilic prostaglandin, 15-deoxy-Δ12,14-prostaglandin J2 (15d-PGJ2), has been show to activate TRPA1 via a mechanism similar to that of MO [172]. In addition, inflammatory sensitization of TRPA1 may underlie some components of inflammatory hyperalgesia (increased sensitivity to painful stimuli), particularly mechanical and cold hyperalgesia [156,173,174]. Thus, TRPA1 is most likely a chemical sensor for injury and inflammation.

### 3.6 Therapeutic Potential

Abundant expression of TRPA1 in DRG and nodose C-fibres innervating airways has recently led to the discovery of the role of TRPA1 in respiratory physiology and pathology [175-177]. Indeed, activation of neurons innervating airways by irritants, oxidants or allergens causes respiratory depression, nasal obstruction, cough and sneezing. Importantly, asthma in many cases is triggered by airborne irritants (such as cigarette smoke components) capable of activating TRPA1 and exciting airway C-fibres. Such C-fibre excitation not only triggers spinal respiratory circuits but also stimulates terminal release of neuromediators such as substance P, neurokinin A, CGRP, and others [178]. Local release of neuropeptides may further enhance airway inflammation (neurogenic inflammation) as well as cause contraction of airway smooth muscles, trigger bronchial oedema, mucus secretion and other asthma-related pathologies [178]. Strikingly, a recent study provided evidence that *Trpa1*-/- mice are much more resistant to airway inflammation and hyperactivity in an allergic asthma model than the wild-type animals. Moreover, treatment of wild-type mice with a TRPA1 antagonist significantly inhibited airway inflammation and hyperactivity [175]. This study highlighted the perspectives of TRPA1 antagonists for treatment of inflammatory diseases of airways and, particularly, asthma. There also may be a potential for targeting TRPA1 in the development of anti-tussive drugs. A different study recently suggested that a TRPA1 antagonist has efficacy in reversing mechanical hyperalgesia induced by inflammation [174].

An unexpected use of pro-algesic thermo-TRP channels in analgesic drug delivery has been found [179]. Using the TRPV1 pore dilation phenomenon it was possible to deliver cell-impermeable lidocaine analogue QX-314 specifically into the TRPV1-positive nociceptors by local co-injection of QX-314 with TRPV1 agonist capsaicin. This manoeuvre allowed local analgesia without motor or tactile deficits normally produced by local administration of cell-permeable lidocaine, which equally affects all type of nerve endings within the injection area. In further development of this approach this group demonstrated that lidocaine itself is capable of TRPV1 activation and, thus, co-application of lidocaine and QX-314 increased efficacy of local anaesthesia by the selective delivery of QX-314 to subset of nociceptors [180]. In a preliminary report the group suggested that TRPA1 can also be used for the local delivery QX-314 to TRPA1-positive nociceptors [181]. This work introduces a new avenue for the design of more selective local analgesics. 

### 3.7 Pharmacology

Activators of TRPA1 were discussed above; TRPA1 channels are inhibited by gentamicin, ruthenium red and gadolinium (all in low micromolar range; reviewed in [122]), although these are all relatively non-specific agents. Despite the fact that TRPA1 is a validated target for neurogenic inflammation, asthma and several types of pain, the number of known selective TRPA1 inhibitors is surprisingly low. Viana and Ferrer-Montiel reviewed available patent databases and found only 14 TRPA1-related patents, of which only five protected TRPA1 inhibitors [182]. Thus, lower alkyl phenols such as (+/-) camphor inhibit TRPA1 with IC_50_ in lower millimolar range [182]. (Z)-4-(4-chlorophynyl)-3-methylbut-3-en-2-oxime (AP18) also block TRPA1 with an IC_50_ of about 3 µM; this compound also showed antihyperalgesic efficacy in behavioural models of inflammatory pain [174]. An AP18-related compound, (1E,3E)-1-(4-fluoropheny)-2-methylpent-1-3-one oxime, blocks TRPA1 with an IC_50_ of 70 nM, which is the most potent TRPA1 blocker known by far [182]. Two related compounds, 2-(1,3-dimethyl-2,6-dioxo-1,2,3,6-tetrahydropurin-7-yl)-N-[4-(propan-2-yl]phenyl) acetamide (HC-030031) and 2-(1,3-dimethyl-2,6-dioxo-1,2,3,6-tetrahydropurin-7-yl)-N-[4-(butan-2-yl]phenyl) acetamide (CHEM-5861528) block TRPA1 with IC_50_s in the range of 4-10 µM [147,182]. HC-030031 was shown to reduce airway inflammation and hyperexcitability in a model of asthma [175].

## CONCLUSIONS

The evidence suggests that these three TRP channels have in common that they are quite broadly distributed (TRPA1 perhaps mostly via sensory nerves), show polymodal stimulation, have activities that are enhanced by redox factors, are permeable to calcium, and are facilitated by elevations of intracellular calcium. There are, however, also important differences.

TRPC5 activity is stimulated by a wide range of agonist at G protein and tyrosine kinase receptors but it is also promiscuous in showing stimulation by a range of additional substances including specific lipids, acid, and metal ions. It has intriguing roles in cellular or sub-cellular movements and is implicated in the control of fear. Agents targeted to TRPC5 or TRPC5-containing channels may be useful in suppressing unwanted tissue remodeling and anxiety. TRPM2 senses hydrogen peroxide but also specific nucleotides and may offer an avenue for development of new agents that suppress stress-related inflammatory disorders, adverse effects of stroke, and degenerative conditions of the nervous system and pancreas. TRPA1 is a sensor for chemical irritants and has additional possible roles in thermo- and mechano- sensation. Most notably, stimulation of TRPA1 excites nociceptive neurons, apparently contributing significantly to physiological pain responses, inflammatory hyperalgesia, neuropathic pain states and irritant responses of asthma. TRPA1 has potential as a target for developing new analgesics and agents that treat asthma.

Based on current evidence it seems unlikely that substantial unwanted effects would arise if specific inhibitors of the channels were administered because disruption of the genes has relatively little effect on the mouse in controlled conditions. There would naturally need to be much more investigation of potential safety concerns however, and there is the additional problem that highly-specific and potent small molecule blockers of the channels are not yet known, perhaps with the exception of TRPA1 agents.

Fig. (**1**) is a concise summary of key features of the three TRP channels, indicating physiological roles, and suggesting conditions in which blockers of the channels might be therapeutically useful. The figure is not exhaustive and so readers are referred to the main text and original articles for more complete information. The channels show common themes in their sensitivities to redox factors and other reactive chemical species, permeability to calcium ions, and stimulation by elevation of the intracellular calcium concentration. Although there is evidence for roles of the channels in quiescent conditions, they may be most functional in adverse conditions, making them potentially attractive as therapeutic drugs targets. The potential for integration with oxidative stress mechanisms of mitochondria may be a fruitful area for further investigation [183].

## Figures and Tables

**Fig. (1):**
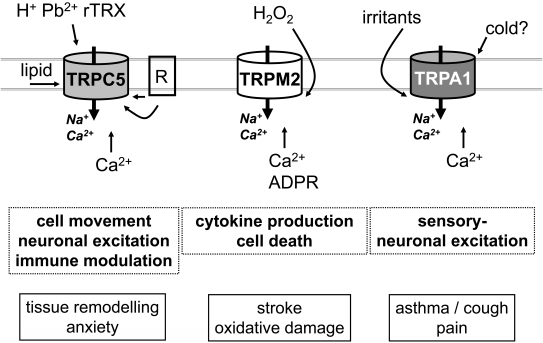
**Example TRP channels of adversity.** Simplified summaries of the TRP channel properties (upper schematics), functions (bold text in dashed boxes) and potential therapeutic opportunities (text in solid boxes). R, receptor; rTRX, reduced thioredoxin; ADPR, adenosine diphosphate ribose. See main text for details.
